# Examination of Traditional Botnet Detection on IoT-Based Bots

**DOI:** 10.3390/s24031027

**Published:** 2024-02-05

**Authors:** Ashley Woodiss-Field, Michael N. Johnstone, Paul Haskell-Dowland

**Affiliations:** 1 School of Science, Edith Cowan University, Joondalup 6027, Australia; a.woodiss-field@ecu.edu.au (A.W.-F.); m.johnstone@ecu.edu.au (M.N.J.); 2 Security Research Institute, Edith Cowan University, Joondalup 6027, Australia

**Keywords:** botnet, Internet of Things, Mirai, detection

## Abstract

A botnet is a collection of Internet-connected computers that have been suborned and are controlled externally for malicious purposes. Concomitant with the growth of the Internet of Things (IoT), botnets have been expanding to use IoT devices as their attack vectors. IoT devices utilise specific protocols and network topologies distinct from conventional computers that may render detection techniques ineffective on compromised IoT devices. This paper describes experiments involving the acquisition of several traditional botnet detection techniques, BotMiner, BotProbe, and BotHunter, to evaluate their capabilities when applied to IoT-based botnets. Multiple simulation environments, using internally developed network traffic generation software, were created to test these techniques on traditional and IoT-based networks, with multiple scenarios differentiated by the total number of hosts, the total number of infected hosts, the botnet command and control (CnC) type, and the presence of aberrant activity. Externally acquired datasets were also used to further test and validate the capabilities of each botnet detection technique. The results indicated, contrary to expectations, that BotMiner and BotProbe were able to detect IoT-based botnets—though they exhibited certain limitations specific to their operation. The results show that traditional botnet detection techniques are capable of detecting IoT-based botnets and that the different techniques may offer capabilities that complement one another.

## 1. Introduction

A botnet is a set of networked devices that have been compromised by a malicious actor (a botmaster) to conduct various activities, such as sending spam or initiating Denial-of-Service attacks. Such compromises often occur due to vulnerabilities in the software running on the devices, leaving said devices amenable to outside control. A device that is assumed into a botnet is known as a bot or a zombie. The actions of the bots are coordinated by a command-and-control (CnC) structure. An individual bot does not necessarily pose a significant threat, but the ability of a botmaster to control a multitude of bots, with their attendant processing power, is a problem of a very different scale. Botnets are commonly used to initiate Distributed Denial-of-Service (DDoS) attacks, spamming, phishing, malware distribution, click fraud, and crypto-jacking [1,2,3,4,5].

Traditional (pre-IoT) botnets would usually propagate by targeting workstations, personal computers, and servers. The first widely known botnets would utilise the Internet Relay Chat (IRC) protocol to send commands to their bots. Later botnets would include those that utilise Peer-to-Peer (P2P) protocols and the Hypertext Transfer Protocol (HTTP), with the former facilitating the construction and use of decentralised botnets and the latter using a pull-based command-and-control approach [1,5,6].

Recently, the advent of the Internet of Things has meant that botnet creators have been particularly agile in leveraging the IoT to spread bots and perform attacks. In stark contrast to their traditional botnet counterparts, this rich environment and confluence of protocols presents new challenges in terms of both the variety of threats posed and techniques for detection [7]. The IoT has been instrumental in the rise of the smart city concept. The diversity of networks, protocols, and devices found in a smart city offers many ideal propagation targets for botnets [8].

Many existing botnet detection techniques are designed for traditional botnets, which raises questions about their effectiveness against more recent IoT-based botnets (given the differences between the two environments). Several botnet detection techniques have been developed based on honeypots or static-signature-based detection techniques. Such techniques may be effective against IoT botnets but would not detect unknown-unknown (zero-day) threats. Some techniques that rely on specific botnet properties (e.g., netflow patterns across bots in compromised networks) have been effective for specific botnets [5,9,10,11,12].

### 1.1. Motivations

This paper, a continuation of the work conducted in [5], describes the reconstruction or acquisition of a number of botnet detection techniques, BotMiner, BotProbe, and BotHunter, followed by experimentation on those techniques to determine their effectiveness on IoT-based botnets.

The experiments conducted involved a number of simulated networks, which included both traditional and IoT-based infection targets. Among the scenarios for experimentation were a number of simulated botnets with different CnC protocols, different rates of infection among the networks, and a variable number of total hosts among the simulated target networks. Two externally acquired datasets, one with traditional bot traffic and one with IoT bot traffic, were also used to further validate the experiments. The goal of the experiments was to test the hypotheses surrounding the strengths and weaknesses of the traditional botnet detection techniques.

This paper seeks to address the potential weaknesses of older botnet detection techniques on IoT-based botnets. The Mirai botnet is a well-known IoT-based botnet that utilised a non-standard bitstream-based CnC protocol and had a propagation approach that did not target entire networks. Among the experimental simulations, non-standard protocols and a varying number of infected hosts were included to determine each of the selected botnet detection technique’s capabilities. Many IoT-based botnets still utilise the IRC protocol, which was also included among the experimental scenarios. The techniques were also tested against traditional botnet simulations to ensure their validity for their intended targets.

### 1.2. Contributions

The contributions of this paper are the evaluation of traditional botnet detection techniques against the following:

- Simulated IoT traffic;
- Established IoT datasets.

The techniques were evaluated with the datasets described above under a light-touch scenario, as used by Mirai, viz., taking over only a few devices per network. This approach was used to increase realism and to increase the difficulty of detection, as the techniques are known to perform well when entire networks are infected. A further refinement, which added to the realism, was the introduction of aberrant devices, i.e., legitimate devices that had gone awry and generated bot-like traffic.

This work differs from previous contributions in that it is an assessment of previously proposed traditional botnet detection techniques, re-applying them to more contemporary threats. Various IoT-based botnet detection techniques have been proposed, as discussed in Section 2.3, which are confronted by a number of common challenges, such as deployment and over-specialisation. This work instead focusses on previously established techniques and attempts to determine whether those techniques are still applicable to modern IoT-based botnets.

This work is a continuation of that conducted in [5]. The key differences between this paper and [5] is the inclusion of BotProbe and BotHunter (as [5] only discusses experiments with BotMiner), the presentation of results (with a focus on true positive rates (TPRs) and true negative rates (TNRs)), and experimentation using externally acquired datasets to further validate the results derived from internal simulations.

### 1.3. Organisation

The remainder of this paper is as follows. Section 2 reviews the relevant background, including other traditional botnet detection approaches not selected for experimentation and contemporary approaches to IoT-based botnet detection. Section 3 describes the traditional bot detection techniques that were experimented on and the hypotheses by which they are assessed. The experimental design, simulation approach, and externally acquired datasets are outlined in Section 4. The experimental results are shown in Section 5. Section 6 discusses the experimental results, outlining how each technique addresses the hypotheses and how they performed on the simulated botnets. Section 7 concludes the work.

## 2. Related Work

### 2.1. Botnet Structures and Operations

Traditional botnets can be categorised by a number of criteria, particularly their CnC structure and the approach by which bots receive their commands from their CnC structure. CnC structures are commonly either centralised or decentralised, with the former being more straightforward to operate and maintain. Bots will either pull commands from the botnet CnC structure (pull-based) or have their commands sent to them from said structure (push-based). Pull-based botnets commonly use the HTTP protocol, whereas push-based botnets commonly (though not exclusively) use the IRC protocol. Among the simulated experimental scenarios, centralised push-based and centralised pull-based botnets are included. A decentralised P2P botnet is included in the externally sourced dataset [5,13].

Botnets have been seen to propagate in a number of different ways. Email attachments, drive-by downloads, and social engineering techniques are some methods by which botnets are allowed to propagate. These can come in the form of worms that exploit software or firmware vulnerabilities to propagate over poorly supported devices or trojans that disguise themselves as legitimate programs but contain backdoors through which a malicious actor can take control [14,15]. More modern and highly automated approaches to botnet propagation include actively seeking out poorly secured devices over telnet or ssh and running dictionary attacks to take over devices with default credentials [7,16,17].

HTTP-based botnets utilise a pull-based and centralised CnC structure. HTTP bots will routinely query the CnC structure to receive commands, as opposed to being issued the commands directly. HTTP-based botnets are notable in that they are typically more difficult to detect than their IRC and P2P counterparts, as they operate using a common protocol. Notable HTTP-based botnets include Festi, Grum, SpyEye, and Zeus. SpyEye and Zeus in particular differ from other bots in that they would primarily be used to propagate themselves and steal user information from their hosts, whereas most botnets are used to attack other targets [5,13,18].

IRC-based botnets utilise a push-based and centralised CnC structure. IRC bots will receive their commands from the CnC structure via an IRC channel. IRC-based botnets make up some of the first botnets to be widely known. Notable IRC-based botnets include GDbot, SDbot, Agobot, and Spybot [13,18]. Many IoT-based botnets also use the IRC protocol (Mirai being a well-known exception) [5,7].

P2P botnets are typically more complex than their centralised counterparts and require expert knowledge to operate. P2P botnets became widely known some time after their IRC counterparts and before HTTP-based botnets. Prominent P2P botnets included Sinit, Phatbot, SpamThru, Nugache, and Peacomm/Storm. The lattermost P2P botnet is the one examined from the externally acquired dataset for this experiment [1,5,13].

IoT-based botnets have risen as a more contemporary threat, with their primary distinction being the devices they target for propagation. IoT-based botnets are able to benefit from a number of advantages not typically available to their traditional counterparts. Many IoT devices are poorly secured not having default credentials changed or not having their firmware updated [7,19]. These devices are also numerous: the number of IoT devices is predicted to reach over 50 billion devices by 2050 [20]. Due to the large number of available devices, botnets can propagate a lot faster than before and can even be selective with the networks and devices they take over. Many IoT devices are designed to remain operational at all times and are not shut down when not in active use, allowing them to be infected and utilised by botnets on demand. With such a large number of devices available to take over and utilise at virtually any time, IoT-based botnets can grow far larger and act far more aggressively than their traditional counterparts [13].

Mirai is a notable IoT-based botnet that was able to infect over 140,000 devices and conduct attacks at speeds over 1 Tbps. Partly based on another botnet, Bashlite, Mirai would propagate via telnet using a dictionary attack with commonly used default credentials. Mirai would use a federated CnC structure, where devices would report potential infection targets once a successful telnet authentication had been made. Mirai would target hosts at random, and as such, would not directly attempt to propagate over entire networks [21]. Other IoT-based botnets include Linux/IRCTelnet, Aidra, The Moon, and Linux/Hydra [22,23].

In addition to the advantages inherent to IoT devices, other factors may also compromise conventional botnet detection techniques. Centralised CnC protocols, for example, are not necessarily constrained to only HTTP or IRC [13]. Any detection technique that focusses on a limited set of protocols will be incapable of detecting a botnet operating over a different protocol. Mirai, for example, used a binary stream to command the bots it suborned [24]. Mirai would usually only control a single device within a targeted network, a deviation from traditional botnets, which would typically aim to take control of as many devices as possible. Mirai’s scanners sought out vulnerable devices randomly. Found devices would be checked against a blacklist, and then a telnet login would be attempted using a list of common username and password combinations. Found devices that can be accessed would then be referred to the malware distribution server. Mirai was enabled to propagate the way it did because of the availability of vulnerable devices to infect. The fact that many IoT devices are also always on also meant that Mirai, and other IoT-based botnets, would be able to retain control of infected devices continuously [7,17,21].

The emergence of IoT-based botnets, in terms of both their greater threat power and their potential ability to evade traditional detection techniques, is the focus of the project being undertaken. By assessing the current ability of traditional botnet detection techniques in the literature, their abilities and the need for further action can be determined. Certain aspects of current techniques can also potentially be applied to IoT-based botnet detection [25].

### 2.2. Traditional Botnet Detection Techniques

BotMiner, BotProbe, and BotHunter are each being investigated to understand their capabilities in IoT-based botnet detection. Other techniques have also been proposed for traditional botnet detection but can be ruled out for IoT-based botnet detection on the basis of their fundamental features. Internal host-based detection techniques are prime examples of traditional botnet detection techniques that cannot be deployed on many IoT devices, as they require significant host resources to operate properly [26].

RB-Seeker is a botnet detection technique that works by examining the temporal and spatial features of Domain Name Service (DNS) redirection activities to identify domains and distinguish botnet queries from legitimate queries using established feature statistics. RB-Seeker is posited to act fast and can be applied to any kind of botnet, regardless of the protocol and CnC structure. However, it is constrained to a limited scope of attack types, redirection, and proxy scams and has limited capabilities on host networks that utilise dynamic DNS [2,27].

BotSwat is a host-based botnet detection technique that examines a given host’s processes that use data from external sources that have been labelled as unreliable. BotSwat operates by curating the internal processes of a host and determining bot activities based on remote control characteristics. Unfortunately, BotSwat is prone to producing false positives in cases where said characteristics are present in legitimate programs. BotSwat would not be suitable for low-powered devices or any devices expected to interact with remote external sources [9,26].

A method of P2P botnet detection using various machine-learning techniques was experimented with by [28]. Experimentation with various machine-learning techniques in botnet detection measured the training time, classification time, detection rate, and error rates. Although Support Vector Machines and Artificial Neural Networks had the highest detection rates, both methods took the longest to train. According to the authors, their training times appeared to make the techniques unsuitable for adaptable online botnet detection [28].

### 2.3. IoT-Based Botnet Detection Techniques

Some initiatives have been developed towards IoT-based botnet detection; however, many appear to either be honeypot approaches, which would not help during an active zero-day threat, or only fit specific scenarios. Refs. [29,30] developed a specialised honeypot and sandboxing technique, respectively, useful for observing and understanding known threats but limited for detecting new threats.

In [31], an approach for detecting anomalous IoT-device behaviour through the use of deep autoencoders was developed. The approach involves training an autoencoder for each IoT device in order to learn its normal behaviour. Then, if the autoencoder fails to reconstruct a snapshot of subsequent activity, that failure indicates anomalous activity. While this technique would potentially operate well on simple single-function devices, it would fail to operate on more complex devices, such as home automation hubs, smart devices, or network infrastructure devices.

A graph-based approach for IoT bot detection was developed in [32]. The approach involves the analysis of Printable String Information graphs to determine stages of bot infection, including scanning, accessing, infecting, communication, and awaiting commands. However, the technique is limited specifically to devices, and the botcode, using Executable and Linkable Format binaries. Furthermore, the technique relies on training with externally sourced datasets to determine what can be classified as benign and malicious. It appears to operate on a similar principle to BotHunter, though with a more limited scope.

In [33], a deep modelling technique for IoT bot detection based on power consumption was developed. The technique was tested on common IoT devices, such as the kind infected by the Mirai botnet, including cameras and routers. Similar to the technique developed in [31], standard operating behaviour for the devices is used to train the technique, which allows anomalous activity to be detected. However, much like the technique developed in [31], this technique would only be applicable to devices with single or few functions, with more complex IoT devices and the supporting infrastructure remaining less protected. Other factors that could potentially affect the power consumption of IoT devices, such as unrelated issues with the power supply, may also produce false positives.

Various IoT-based botnet detection techniques have involved utilising multiple learning models, including Random Forest variants, Logistic Regression, Decision Tree variants, Gaussian Naïve Bayes, eXtreme Gradient Boost, and K-Nearest Neighbour. These techniques include BotStop [34] and ELBA-IoT [35], among others [36,37]. Common concerns among some of these approaches are deployment challenges and the impact on low-powered devices.

It appears that outside of honeypots, Intrusion Detection Systems (IDSs), and sandboxes, techniques developed specifically for IoT devices are limited in scope in terms of which IoT devices can be monitored effectively. In the case of simple, single-function devices, techniques such as that in [31,33] may prove adequate; however, many IoT devices perform multiple and often complex tasks or are subject to complicated scenarios. One of the datasets sourced for the experiment undertaken contains device activities for an Amazon Echo, for example, which would likely produce an alternate snapshot of activity each time it is assessed, as well as exhibiting varying power usage.

### 2.4. IoT-Fog Networks

Cloud computing is a technological paradigm that operates on providing on-demand network access to shared computing resources that can be provisioned and deployed with minimal effort. Whereas the IoT involves the use of many distributed and interconnected devices that continually adjust their functionality based on the deployment environment, cloud computing involves remotely adjusting resources in order to meet functionality needs. The goals of cloud computing and the IoT conflict, as one is about adjusting provisioned resources and the other is about utilising resources to adjust functionality [38,39].

Edge computing encompasses technologies that perform computations at the edge of a network, extending cloud computation capabilities to be closer to the network’s IoT devices. Fog computing is an advanced implementation of edge computing that brings the delegation of tasks closer to the IoT devices in a more distributed approach, with the aim of reducing issues pertaining to latency and bandwidth [38,39,40].

Fog computing allows for IoT and cloud interoperability but presents security challenges, particularly with regard to maliciously controlled IoT devices attacking services at the fog layer. Software-defined networking (SDN) provides flexible network programmability and management. The coordination of fog and IoT devices through SDN mitigate some of the security concerns introduced with IoT-Fog networks, including the deployment of detection techniques, such as those discussed in this work and related work [41,42,43].

## 3. Proposed Approach

The approach in this work involved acquiring or reconstructing the selected botnet detection techniques, BotMiner, BotProbe, and BotHunter, and applying them to simulated botnets. A number of hypotheses are outlined in Section 3.1 and are used to determine the effectiveness of the chosen botnet detection techniques. Each botnet detection technique was analysed in terms of its approach and reported capabilities. Section 3.2 discusses each of the techniques, how they work, and how they were acquired or reconstructed. Differences between reconstructions and their respective original works and any limitations with regard to the experiments are also discussed.

### 3.1. Hypotheses

For each of the traditional botnet detection techniques experimented on, the following hypotheses were constructed:

- H_1_ The botnet detection technique is not capable of detecting IoT-based botnets.
- H_2_ The botnet detection technique is capable of detecting traditional botnets.
- H_3_ The botnet detection technique is capable of identifying all devices infected with traditional botcode.
- H_4_ The botnet detection technique is capable of identifying all devices not infected with traditional botcode.
- H_5_ The botnet detection technique is not capable of identifying all devices infected with IoT-based botcode.
- H_6_ The botnet detection technique is not capable of identifying all devices infected with IoT-based botcode.

H_1_ and H_2_ pertain to each botnet detection technique’s ability to detect botnets in a given network. For a technique to be considered as having detected a botnet, it only has to correctly identify at least one bot in an infected network—regardless of any false reports or whether a number of bots have still gone undetected. If a technique is able to detect a bot, it has effectively detected a botnet. The hypotheses assert that each traditional technique can detect traditional botnets but cannot detect IoT-based botnets.

H_3_ and H_5_ pertain to detection techniques detecting all bots in an infected network. Unlike with H_1_ and H_2_, these hypotheses test the detection techniques’ ability (or lack thereof) to correctly identify all instances of infection in a network at a given time. H_3_ asserts that traditional techniques can detect all traditional bots, whereas H_5_ asserts that those techniques cannot do the same for IoT-based bots.

H_4_ and H_6_ pertain to the detection technique’s ability to correctly identify non-infected hosts and avoid false positives. If a given technique can avoid mislabelling one or more non-infected hosts as bots, then it can be considered to have identified all non-infected devices. H_4_ posits that the traditional techniques can avoid false positives in traditional networks, whereas H_6_ asserts that the same cannot be said for IoT-based networks.

Each technique will be assessed independently with each of these hypotheses in mind. H_1_ and H_2_ constitute the main focus as to the capabilities of traditional botnet detection techniques and whether or not they can detect IoT-based botnets. H_3_ through H_6_ pertain to the finer points of each technique’s capabilities with regard to TPR and TNR—allowing each technique to be compared to the other two and determining each of their strengths and weaknesses.

### 3.2. Botnet Detection Techniques

#### 3.2.1. BotMiner

BotMiner is a botnet detection technique that collects netflow data and IDS alerts, clusters both of these based on a number of features, and performs a cross-clustering analysis to determine whether or not a given host is a bot. BotMiner will cluster alerts based on their categorisation; this could be based on pre-determined categories or on alert signature categories, for example. For netflow clustering, BotMiner uses a number of features, such as those based on time and throughput, and X-Means clustering to determine common patterns of network activity. BotMiner then uses an established threshold and a cross-clustering calculation to render a determination on a given host. BotMiner is protocol-independent and non-invasive: it can be used to detect any kind of botnet and does not directly interfere with network operations. However, some limitations may include its reliance on IDS alert signatures and its design being based on the assumption that botnets will propagate entire networks over individual hosts [11].

As BotMiner (as well as BotProbe) has no publicly available implementation, a reconstruction of it had to be made for experimentation. The analysis and reconstruction of BotMiner were performed and discussed by [25], with experimentation conducted on a number of scenarios (the same presented in this paper) by [5]. BotMiner’s design is based on the premise that botnets will infect many devices on the same network and that those infected devices will exhibit common patterns of behaviour that can be identified as bot activities. BotMiner operates by clustering IDS signature alerts and netflows and then performing a cross-clustering calculation on those clusters. BotMiner is made up of five main components: the A-Plane Monitor, C-Plane Monitor, A-Plane Clustering, C-Plane Clustering, and Cross-Plane Correlation. The A-Plane components pertain to alert collection and clustering, whereas the C-Plane components pertain to netflow collection and clustering [5,11].

The A-plane components of BotMiner uses alert categories to cluster Snort IDS signature alerts. The original technique provides scan activity, spam activity, and binary downloads as examples of alert categories that the A-Plane clustering component clusters by. The technique also uses a statistical scan anomaly detection engine to find and categorise scan activities. However, the reconstruction differs from the original for a number of reasons. The categories provided by the original work are somewhat arbitrary and do not necessarily cover the full scope of botnet behaviours. Scan and spam activities do potentially cover a number of botnet propagation and attack strategies but miss a number of common and contemporary behaviours, such as DDoS attacks (which can only loosely be described as spam activity, if at all) and crypto-jacking. CnC communications are also not covered in the original technique’s category examples. The reconstruction therefore opted to categorise alerts based on the category assigned by the Snort IDS, which proved to be effective [5,11,25].

The C-plane components of BotMiner collect and cluster UDP and TCP netflows, with clustering performed based on a number of features. The original technique utilised a tool called fcapture, whereas a custom program was built for the reconstruction.

Flows are collected from traffic based on their source IP and port, destination IP and port, protocol, and time. Additional features collected include the duration of the flow and the volume of bytes and packets going in both directions for the flow. Based on the source IP, source port, destination IP, destination port, and protocol, these flows are then grouped into C-Flows. These C-Flows are then split over thirteen time intervals, and sample distribution features for flows per hour (fph), packets per flow (ppf), average bytes per packets (bpp), and average bytes per second (bps) are recorded, producing a 52-feature dataset (each sample distribution feature for each interval). The means and variances of the fph, ppf, bpp, and bps are then also determined over the thirteen time intervals, resulting in an eight-feature dataset. X-Means clustering is conducted to determine the best K value using the 8-feature dataset, with the best K value then being used on the 52-feature dataset to produce the final netflow clusters for each given C-Flow [5,11,25].

Once alert and netflow clusters have been established, each identifiable host to which the clusters can be attributed are put through a cross-clustering process. This process utilises the calculation present in Equation (1). Equation (1) utilises the sequence of hosts within both alert and netflow clusters, with the former having the capability to be weighted. For all unique alert cluster combinations, excluding those where the alert type is the same (described in terms of i and j in sequence in Equation (1)), the weights of the activity types (which, for this implementation, is 1) are multiplied between those of both alert types. That is then multiplied by the cardinality of the intersection of hosts within the two alert clusters i and j over the cardinality of the union of hosts within the two clusters i and j. The sum of the first sequence is then added to the sequence of the alert clusters and the netflow clusters (described in terms of sequences i and k), where the weight of activity type i (always 1 for this implementation) is multiplied by the cardinality of the intersection of hosts in alert cluster i and netflow cluster k over the cardinality of the union of hosts in alert cluster i and netflow cluster k. The sum of the two sequences produces the final bot-score.

Weights are not utilised in the reconstruction or the original work but are available should a method for implementing them ever become available. The calculation renders a bot-score for each host, which is compared to an established threshold (in this case, 1, as was set in the original work). If the bot-score is equal to or greater than the threshold, the host is designated as a bot.

(1) $$s\left(h\right)=\sum_{\begin{array}{c} i,j\\ j>i\\ t\left(A_{i}\right)\neq t\left(A_{j}\right) \end{array}}w\left(A_{i}\right)w\left(A_{j}\right)\frac{|A_{i}\cap A_{j}|}{|A_{i}\cup A_{j}|}+\sum_{i,k}w\left(A_{i}\right)\frac{|A_{i}\cap C_{k}|}{|A_{i}\cup C_{k}|}$$

where *A* is the sequence of hosts within alert clusters; *C* is the sequence of hosts within flow clusters; and *w* is the activity-type weight.

#### 3.2.2. BotProbe

BotProbe is a botnet detection technique that uses a number of approaches to detect bots by eliciting deterministic responses. Specifically, BotProbe has a session-replay-probing component that operates by spoofing the address of a suspected CnC server and replays recorded commands to a suspected bot. If the suspected host responds in a deterministic manner consistent with the previously recorded response traffic of the suspected bot command, that host is determined to be a bot. BotProbe does have some limitations in terms of applicability; its original implementation operates on IRC and instant message bots specifically, and by design, it can only detect push-based bots [12].

The analysis and reconstruction of BotProbe were discussed by [44], with further improvements made for the experiments presented in this work. BotProbe operates on the premise that botnet command responses are deterministic, that when a bot command is received, the following activity from that bot will be consistent with that command every time. The original BotProbe work consisted of a feasibility study consisting of several techniques, including explicit challenge response testing, session replay probing, session byte probing, client replay probing, man-in-the-middle probing, and multiclient probing. The work conducted by [44] and in this experiment focusses specifically on session replay probing [9,12].

Session replay probing involves spoofing the address of a suspected CnC server and replaying captured application commands to a suspected bot. If the suspected bot responds in a consistent and deterministic manner, it is reported to be a bot responding to CnC orders [12].

The reconstruction of BotProbe was made in two components: one component filters for potential bot commands, and the other conducts the probing using commands found with the filter. This approach allows the probing component to potentially be re-used with other filters. The filter component can also be used on pre-recorded traffic, allowing for greater applicability—though the probing component can still only operate in live environments. The filter component for the experiments presented in this work utilises an IRC-based filter. Suspected bot commands, and their responses, are relayed to the probing component, which spoofs the command a configurable number of times to the suspected bot. The following traffic of the suspected bot is then recorded for up to a specified number of frames within a specified timeframe. The original command response and the probe responses are compared, where the constitution and ordering of the response frames are considered: if the responses match, the suspected host is reported as a bot.

Some limitations in BotProbe’s design excluded it from a number of experiments presented in this work. The reconstructed filter was designed specifically for the IRC protocol, meaning that only IRC-based botnet simulations could be used. Experimentation with BotProbe was still considered, as a number of IoT-based botnets do use the IRC protocol for their CnC, including Linux/IRCTelnet, Linux/Hydra, and Aidra [7]. Another limitation of BotProbe that impacted experimentation was that BotProbe, specifically the probing component, can only operate on live traffic. While the filtering component can still be applied to pre-recorded traffic, the probing component requires a live environment, as its operation is dependent on host responses. This meant that BotProbe could not be applied to externally sourced traffic recordings as the other techniques were.

#### 3.2.3. BotHunter

BotHunter is a botnet detection technique that uses lifecycle modelling to detect potential bots. Similar to BotMiner, BotHunter utilises IDS signatures to collect alerts. Those alerts are then applied to one of several bot lifecycle models. Once the model has completed, to a pre-determined extent, the collection of alerts over time, the host to which the alerts pertain is designated as a bot. BotHunter shares some strengths with BotMiner in that it is protocol-independent and can be applied, in theory, to both live environments and packet captures. However, it shares some limitations with BotMiner, being somewhat constrained to IDS signatures, as well as having its own unique limitations, such as being constrained by its limited number of lifecycle models. Unlike BotMiner and BotProbe, which had to be reconstructed based on the literature for this work, BotHunter has a publicly available distribution, which was used for experimentation [10].

BotHunter’s publicly accessible implementation can be found at http://www.bothunter.net/, accessed on 1 December 2023. It is packaged with the MetaFlows sensor, a network IDS implementation that utilises multi-session alert event reports for threat detection. While the MetaFlows sensor is a licensed product, the BotHunter implementation is available for non-commercial use. BotHunter can be installed via a plug-and-play virtual machine or installed on a host running the CentOS operating system.

BotHunter’s design does not necessarily require it to be deployed on live traffic, but the implementation does not allow for its direct application to previously made traffic recordings. In order to test BotHunter in the same environments as the other techniques, as well as on externally sourced traffic recordings, tcpreplay was used to replay the traffic. As BotHunter does not directly interact with any of the host devices, unlike BotProbe, this method of replay was an acceptable approach to emulating a live environment.

## 4. Performance Evaluation

The experiments undertaken for the selected botnet detection techniques were previously conducted in [5], specifically on BotMiner. The same experiments were conducted for this paper but were applied to BotProbe and BotHunter in addition to BotMiner—with the additional inclusion of externally sourced datasets to further validate the results, where possible.

The primary purpose of the experiments undertaken was to determine the applicability of traditional botnet detection techniques to IoT-based botnets. To this end, a number of IoT-based botnet simulations were developed for the selected botnet detection techniques, BotMiner, BotProbe, and BotHunter, to be applied to. A secondary purpose of the experiment was to validate each acquired botnet detection technique, particularly the reconstructions of BotMiner and BotProbe, and ensure that each technique was able to achieve what it was originally designed for. To this end, traditional botnet simulations were also developed [5].

### 4.1. Simulations

Four kinds of botnets were simulated in two types of networks, with a number of variations in terms of the number of network hosts, the number of infected devices, and the presence of aberrant activities included to further explore each technique’s capabilities. The two types of networks included a simulated traditional network made up of simulated workstations made to emulate some human-like behaviours with its network traffic and a simulated IoT network with a more automated traffic simulation utilising IoT protocols. Each of these two networks had two kinds of botnets applied, distinguish primarily by their CnC protocols and patterns [5].

To further test the detection techniques’ capabilities, variations of the simulated networks were assigned a device that exhibited aberrant behaviour. This aberrant behaviour would produce traffic similar to the attacks committed by the compromised hosts—taking the form of a high volume of Internet Control Message Protocol (ICMP) pings to a given host (albeit internally as opposed to the actual attack behaviours external targets). This aberrant behaviour was included in order to test each technique’s specificity, i.e., its ability to properly identify legitimate hosts and avoid false positives. Whereas networks without an aberrant host represented a network operating as expected, the presence of an aberrant device represented a misconfiguration or device failure that could be misunderstood as bot activity. One aberrant host was included per scenario in order to observe the difference between a network with and without such activity [5].

Of the two traditional botnets simulated for the experiments, one was a pull-based botnet using HTTP as its communication protocol, and the other was a push-based botnet that used the IRC protocol. The HTTP bots would periodically request commands from an HTTP server and conduct their attacks accordingly. The IRC bots would receive their commands from an IRC server. The former would be constrained to a fixed command schedule, whereas commands from the latter could be issued on demand. Both simulated botnets would run simulated DDoS attacks using mass ICMP pings against a simulated victim web server for a duration specified by the command parameters [5].

The simulation environments were created using a number of virtual machine hosts on an ESXi hypervisor. Botnet activities were fully simulated using infected hosts’ designated virtual networks with commands sent from a botmaster and attacks directed at simulated web servers, each also on its own virtual network. Background traffic for both traditional and IoT environments were produced using a scalable Scapy-based solution where HTTP and Advanced Message Queue Protocol (AMQP) packets were emulated. Traffic recordings of these environments were then used for each botnet detection technique, with BotProbe also being applied to a live environment.

For the traditional network simulations, all hosts, including the infected hosts, produced conventional HTTP traffic to present a realistic environment for the experiment. A number of variations based on the total number of hosts, the number of infected hosts, and the presence of aberrant activity were recorded from the traditional network simulations. The recorded experimental scenarios included networks with the following devices:

- Ten total devices or one hundred total devices;
- One, three, or no infected devices;
- One or no aberrant device.

A total of 20 variations, of which 5 generations were created, were produced for the traditional experimental scenarios for both simulated traditional botnets applied.

Of the two IoT-based botnets simulated, both utilised push-based CnC structures. One utilised a custom Transmission Control Protocol (TCP)-based bitstream (BS) communication protocol and heartbeat similar to the Mirai botnet. The BS protocol operates similarly to the original Mirai approach and represents the non-standard and esoteric nature of Mirai’s communication approach. The heartbeat was also included to further distinguish the botnet’s approach from the more common IRC-based CnC and represent Mirai’s CnC patterns. The other simulated IoT-based botnet utilised the IRC protocol, being effectively the same in operation as its traditional counterpart aside from the hosts that it would infect. The simulated IoT-based botnets would conduct the same attacks as their traditional counterparts: ICMP-based DDoS attacks [5].

**Figure 1:**
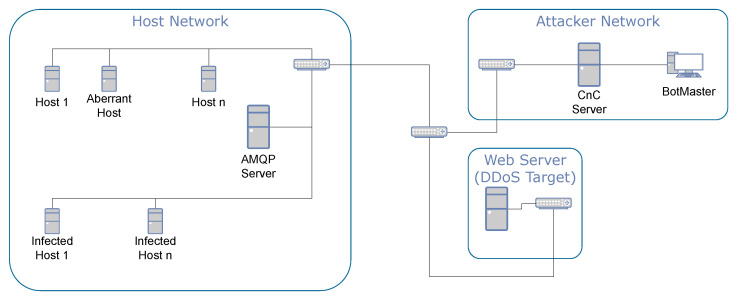
Network diagram of IoT-based botnet simulation, including the infected IoT network, attacker network, and DDoS target network.

The IoT-based network simulations featured the use of an AMQP message broker server and a number of simulated IoT devices using the AMQP protocol. AMQP is an IoT protocol that sends messages through queue exchanges, which are then consumed by subscribed workers [45]. This acted as realistic traffic for the simulated IoT network. Shown in Figure 1 is the network diagram for the IoT-based botnet simulations, including the host network, attacker network, and DDoS victim. The traditional simulations have a similar structure but without the AMQP server on the host network and with a number of additional web servers that they interact with outside of the host network to represent human-like web activities [5].

As with the traditional network simulations, the IoT network simulation recordings included a number of scenarios based on the total number of hosts, the number of infected hosts, and the presence of aberrant activity. Whereas the total number of hosts for the traditional networks only included scenarios with 10 and 100 total hosts, the simulated IoT networks also included scenarios with 500 total hosts to model the scale of IoT networks. The resulting scenarios included networks with the following devices:

- Ten total devices, one hundred total devices, or five hundred total devices;
- One, three, or no infected devices;
- One or no aberrant device.

Thirty variations, of which five generations were created, were produced for the IoT experimental scenarios for both simulated IoT-based botnets applied.

The network variations of both IoT and traditional networks produced a total of 50 environment variations. The detection techniques (with the exception of BotProbe) were applied five times to each scenario, producing a 250-row dataset, inclusive of all four simulated botnets for each technique. The results were recorded based on the number of true positives, false positives, true negatives, and false negatives. The mean TPR and TNR of each technique over all repeated scenarios were used to determine each technique’s capabilities across different scenarios and in comparison to one another [5].

### 4.2. Externally Acquired Datasets

To further validate the results derived from the simulated botnet infections, two external datasets were derived from externally sourced traffic captures. Both a traditional botnet infection dataset and an IoT-based dataset were acquired from [28] and [46] respectively. The externally sourced traffic features instances of real botnets, Storm and Mirai, alongside benign network traffic. While these traffic captures do not exhibit the experimental reliability and general representations provided by the simulations, they do present a more realistic and externally valid demonstration of BotMiner’s capabilities. Due to the limited amount of traffic available from the external datasets, only one scenario that could be directly compared to the simulated results was produced for each set. The scenario developed for both external datasets included 10 total hosts, 1 of which was infected.

The traditional botnet dataset was sourced from the ISOT dataset developed for the experiments undertaken in [28] and provided by the University of Victoria. Bot traffic included within the dataset included that from Zeus, Waledac, and Storm. The format of the dataset was presented within a single packet capture (pcap) file, spanning several months. For the BotMiner experiment, a section of that pcap hosting the Storm botnet was extracted. BotMiner was set to examine the suspected host and nine benign hosts to produce the results. The benign hosts feature those recorded from the day-to-day use of an enterprise network, including web, email, and media-streaming activities [28].

Storm is a P2P botnet known to use the Overnet P2P protocol for its CnC communications. The Storm botnet was primarily designed to send out spam, but its modularity provided it with the ability to switch capabilities, such as performing DDoS attacks. Notably, Storm’s CnC architecture appears to follow a federated pull-based approach, where commands, targets, and updates are pulled by the bots from designated hosts [6,47]. In addition to providing external validity to the experiment, the external dataset also provides insight into BotMiner’s capabilities on a decentralised P2P botnet, a scenario not covered by the simulation that examines centralised botnets.

The IoT botnet dataset was sourced from the IoT-23 dataset developed by the Stratosphere Lab at the Czech Technical University [46]. The dataset includes multiple pcaps of IoT-based botnet traffic alongside some captures of benign IoT traffic. The dataset contains different captures for each recorded host. Measures were undertaken to combine the captures prior to applying the BotMiner detection technique. Only three benign host captures are provided by the IOT-23 dataset; therefore, different time periods from the captures were adjusted to represent a number of different hosts of the same kind of device. A Mirai capture was chosen as the infected host for the experiment, and from the benign captures, nine hosts were derived. The benign hosts include an Amazon Echo, a Phillips Hue device, and a Somfy door lock. The addresses of the Phillips Hue and the Somfy door lock were modified (across different time periods) to represent six additional devices, three additional hosts each. The Amazon Echo was left as a single host. This external traffic set would allow for a real instance of Mirai to be observed while also presenting a variety of IoT devices. In contrast to the simulated dataset, the devices present are more heterogeneous and represent a smart home deployment, whereas the simulation is more representative of a homogeneous industrial deployment [46].

## 5. Experimental Results

BotMiner, BotProbe, and BotHunter (the former two being reconstructions, the lattermost being the original technique) were applied to each simulated network and external dataset—with some exceptions for BotProbe. Due to how BotProbe operates, it was only applied to the simulated botnets utilising the IRC protocol (both traditional and IoT-based) and could not be applied to the external datasets.

Each scenario, differing in terms of the botnet type, total number of devices, number of infected devices, and presence of aberrant activity, was run five times. The results are presented with the mean TPR and TNR of those scenarios among the five runs. The result tables include the results from the traditional scenarios and the IoT scenarios for each botnet detection technique—a total of six tables. The columns of each of those tables include the type of botnet CnC protocol, the total number of devices, the total number of infected devices, the total number of aberrant devices, the TPR, and the TNR. An additional two tables describe the results of BotMiner and BotHunter on the externally sourced datasets. The columns in these tables include the type of botnet (IoT or traditional), the total number of devices, the number of infected devices, the number of true positives, the number of false positives, and the number of true negatives. Each table presents the capabilities of each botnet detection technique in terms of sensitivity and specificity for each experimental scenario.

The results for BotMiner were previously recorded by [5], which can be compared to the results of BotProbe and BotHunter for the same scenarios and discussed in terms of the presented hypotheses for this work.

### 5.1. BotMiner

As mentioned, previous work on BotMiner was conducted by [5]. The results presented here are summarised to present the mean TPR and TNR of each run scenario.

#### 5.1.1. Traditional Botnets

Table 1 shows the mean TPR and mean TNR for each traditional scenario where BotMiner was applied. Immediately apparent is that BotMiner was able to detect every infected host across all scenarios, in line with expectations for the traditional simulations. The protocol used by the botnet, the total number of devices, and the number of infected hosts do not appear to have any impact on BotMiner’s ability to detect bots. BotMiner is also able to detect all bots regardless of aberrant activity. However, when aberrant activity is present, BotMiner appears to be prone to false positives. In all scenarios recorded where aberrant traffic was present, BotMiner incorrectly designated a non-infected host as a bot. This includes the fact that whenever there is no botnet activity, if aberrant behaviour is present, BotMiner will incorrectly identify non-infected hosts as bots.

**Figure 2:**
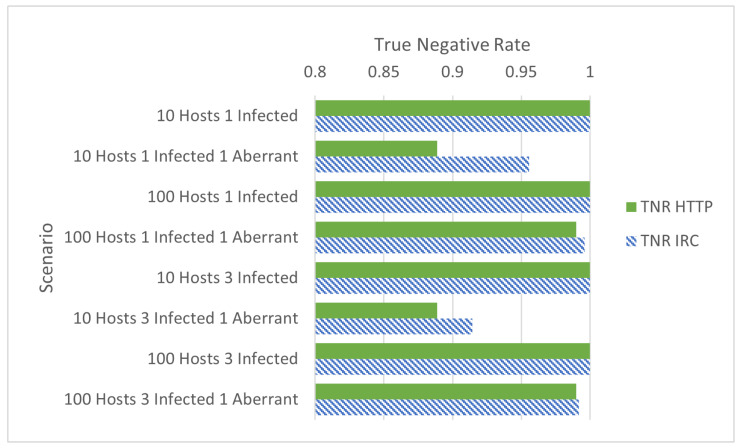
True negative rates of Table 1, grouped by total hosts, infected hosts, and aberrant hosts.

Illustrated in Figure 2 are the TNR rates between HTTP and IRC botnets. Immediately apparent is that when aberrant activity is not present, the results are the same with a TNR of 1—no false positives. However, the TNRs in the HTTP and IRC botnet scenarios differ when aberrant activity is present: BotMiner has a greater TNR when applied to scenarios running IRC-based botnets. Although each IRC scenario had at least one instance where BotMiner produced false positives, a number of runs (of which each scenario had five) among those scenarios successfully labelled all non-infected hosts correctly—accounting for the greater TNR compared to the HTTP scenarios. These differences were also presented by [5].

#### 5.1.2. IoT-Based Botnets

Shown in Table 2 is the mean TPR and mean TNR for each IoT scenario where BotMiner was applied. As happened in the traditional scenarios, and contrary to expectations, BotMiner was able to detect all bots for all scenarios. Also as in the traditional scenario, BotMiner did produce false positives when aberrant activity was present. However, unlike the traditional scenarios, some scenarios with aberrant activity still maintained a mean TNR of 1—meaning that BotMiner effectively performed better than it did with the traditional scenarios.

**Figure 3:**
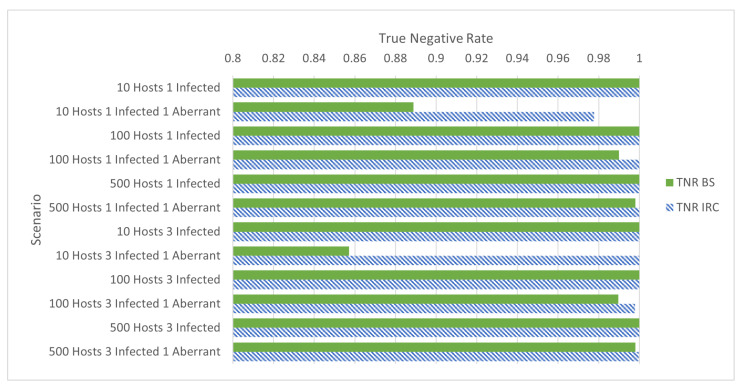
True negative rates of Table 2, grouped by total hosts, infected hosts, and aberrant hosts.

Shown in Figure 3 are the TNR rates of all IRC and BS compared to one another. Similar to the traditional scenarios, all scenarios without aberrant activity have an identical TNR. And where aberrant activity is present, the IRC scenarios resulted in a greater TNR; BotMiner operates better on IRC-based botnets regardless of whether the device is a traditional or IoT botnet target. As mentioned, some aberrant activity scenarios where IRC was used as the CnC protocol produced a TNR of 1—no false positives. It would appear that, for these scenarios, BotMiner performed better on IoT-based botnets than traditional botnets when the same CnC protocol was used.

#### 5.1.3. External Datasets

Shown in Table 3 are the results for BotMiner applied to both external datasets. For the traditional scenario, BotMiner performed as expected; it detected the bot and reported no false positives. For the IoT scenario, BotMiner detected the bot, as it was able to do with the simulations and contrary to the hypotheses, but also produced a number of false positives. These false positives can likely be attributed to aberrant behaviours within the externally sourced datasets that triggered the IDS-based component of BotMiner.

### 5.2. BotProbe

As mentioned, BotProbe has some limitations that excluded it from some of the experiments. Nonetheless, BotProbe was still able to be applied to both a traditional and IoT-based botnet simulation. It should be noted that BotProbe had to be applied to live traffic during its probing stage, meaning that the traffic it was applied to was not necessarily the same as that to which BotMiner and BotHunter were applied. However, the filtering component of BotProbe was able to be applied to pre-recorded traffic. The network configurations were also the same between relevant scenarios, so comparisons can still be made between the results of other techniques, with the extraneous variables caused by the live traffic in mind (consisting mostly of alternative request patterns and timings among the recordings). The results for BotProbe when no bots were present are not included in the tables; those results were uniform, and there were no instances of false positives.

#### 5.2.1. Traditional Botnet

Table 4 shows the results of BotProbe applied to a traditional botnet utilising the IRC protocol. BotProbe was able to detect all bots when only one infected device was present per scenario. However, when multiple infected devices were present, BotProbe did not detect all bots. In scenarios where multiple bots were present with ten total hosts, BotProbe failed to detect any bots with a TPR of 0. When 100 total hosts were present, BotProbe was able to detect a number of bots but was still not able to achieve a TPR of 1. Although BotProbe’s capabilities diminished when multiple infected devices were present, at no point did BotProbe produce false positives: all scenarios produced a TNR of 1.

#### 5.2.2. IoT-Based Botnet

Shown in Table 5 are the results for BotProbe applied to an IoT-based botnet utilising the IRC protocol. In terms of TNR, the results are identical to the equivalent traditional scenarios—a TNR of 1 for all scenarios. As with the traditional results, BotProbe was not able to detect all bots when multiple bots were present, while still detecting each bot in the single bot scenarios. Unlike the traditional scenarios however, there were no instances where BotProbe produced a mean TPR of 0 when applied to the IoT scenarios. Although BotProbe exhibited similar patterns of behaviour, no false positives and a diminished detection rate when more bots were present, it did overall perform better than it had on the traditional scenarios.

### 5.3. BotHunter

BotHunter was unique among the chosen detection technique in that the original implementation of BotHunter was used for experimentation instead of a literature-based reconstruction. The results for BotHunter are presented slightly differently from the other techniques in order to highlight its unique functionality and how it performed between different botnets—particularly with consideration to the IRC protocol. BotHunter has an IDS component which populates the botnet lifecycle model it uses for detection, the TPR of that IDS component is also reported in the tables to highlight instances where BotHunter appeared to have partly worked, even when a full detection had not been made. As with BotProbe, results for BotHunter without any infected hosts are not shown for the same reason—there were no false positives or differences between scenarios when no bots were present.

#### 5.3.1. Traditional Botnets

Shown in Table 6 are the results of BotHunter as applied to traditional scenarios. BotHunter was unable to detect any of the bots. The IDS component of BotHunter was able to produce alerts when applied to IRC-based botnets but was unable to make a full detection using its lifecycle models. It had no apparent effect of HTTP bots which went completely undetected. No false positives were produced but the TPR was consistently 0 across all scenarios.

#### 5.3.2. IoT-Based Botnets

Shown in Table 7 are results that are, where comparable, identical to those in Table 6—no complete detections, and some detections made when the IRC protocol was present. As occurred with the HTTP-based traditional bots, the BS-based IoT bots went completely undetected by BotHunter. No false positives were made, but the TPR was 0 across all scenarios.

#### 5.3.3. External Datasets

The results produced with BotHunter applied to the simulated networks indicated poor performance. It is possible that the simulated scenarios did not account for certain conditions that would have allowed BotHunter to operate that real-life scenarios may have provided. However, the results shown in Table 8 appear to reinforce the findings established in the simulations: BotHunter was unable to make any detections in both the externally sourced IoT and traditional scenarios.

## 6. Discussion

### 6.1. Addressing Hypotheses

Each hypothesis was designed to test each traditional botnet detection technique to determine its capabilities on both IoT-based and traditional botnets. The hypotheses were constructed with the assumption that the techniques would be able to correctly identify all bots and non-bots in traditional networks while not being able to do the same for bots in IoT networks. Three capabilities were tested with these hypotheses for IoT and traditional scenarios: the ability to detect botnets, the ability to identify all bots, and the ability to identify all non-bots. Certain nuances among the results are also apparent, though not necessarily addressed by the hypotheses, such as the tendency for the techniques to perform better on IRC-based botnets and BotProbe’s diminished performance when applied to multiple infections. The outcome of each hypothesis pertaining to each botnet detection technique is shown in Table 9.

BotMiner met expectations in terms of detecting bots in traditional scenarios, and it was able to detect all botnets and all bots: H_2_ and H_3_ were accepted. BotMiner also defied expectations by doing the same for IoT-based botnets. H_1_ and H_5_ were rejected, as BotMiner performed better than expected when it came to detecting bots. However, BotMiner was prone to producing false positives in certain cases, leading to H_4_ being rejected and H_6_ being accepted. BotMiner was unable to correctly identify all non-infected hosts. However, even though H_6_ was accepted, it must be noted that, overall, BotMiner produced fewer false positives in comparable IoT scenarios than it did in the equivalent traditional scenarios—suggesting that BotMiner might have performed better on the more contemporary threat than that which it was designed to address. Although BotMiner has some limitations in the form of over-sensitivity, the network being IoT-based does not appear to have any additional negative impact on its performance.

BotProbe was able to correctly identify all non-infected hosts, and no false positives were produced across all scenarios, both IoT and traditional. This means that it met expectations for the handling of non-infected devices in traditional networks while defying them for IoT networks. H_4_ and H_6_ were accepted and rejected, respectively, and no differences could be identified in terms of false positives between the traditional and IoT scenarios. BotProbe was able to detect bots across a number of scenarios, both traditional and IoT, leading to H_1_ being rejected and H_2_ being accepted. However, BotProbe was not able to detect all bots and had a diminished performance when multiple infected devices were present. Therefore, BotProbe did not meet the expectation given by H_3_, leading to that hypothesis being rejected and H_5_ being accepted. Although H_5_ was accepted, it should be noted that, while two of the traditional scenarios that BotProbe was applied to resulted in a mean TPR of 0, the same scenarios but on IoT networks had a mean TPR greater than 0. BotProbe appears to have performed better with the IoT-based scenarios overall, having detected at least one bot in every scenario.

BotHunter was unable to meet expectations on traditional networks while effectively performing as expected on their IoT counterparts. BotHunter did not detect any bot activity among any of the scenarios, leading to H_2_ being rejected and H_1_ being accepted—the opposite result when compared to the other two techniques. In line with that, H_3_ was rejected and H_5_ was accepted. Technically, BotHunter did not produce any false positives, meaning that H_4_ and H_6_ were accepted and rejected, respectively. Unlike with BotProbe, however, this does not appear to be a result of high specificity but rather a total lack of sensitivity.

### 6.2. Detection Technique Performance

BotMiner had the highest and most consistent mean TPR across all scenarios, successfully detecting all bots in all simulated (and externally recorded) networks. BotMiner did also produce the largest number of false positives among the techniques, however, and was prone to making incorrect assessments whenever devices exhibited aberrant behaviour that could be misconstrued by the technique as malicious. BotMiner’s TNR was greater whenever a botnet utilising the IRC protocol was present, which appears to have allowed BotMiner to make a more accurate distinction between bot and non-bot activities. This apparent pattern of behaviour had a greater impact on IoT-based networks, where BotMiner was able to achieve a mean TNR of 1 in scenarios where it was unable to do so for its traditional counterparts.

BotProbe was able to successfully detect bot activities in almost all scenarios, with only two traditional scenarios where BotProbe had a mean TPR of 0. BotProbe failed to detect all bots in scenarios where more than one host was infected. It appears that the probing technique was unable to properly assess the probe responses to determine whether a given host was infected. When only a single infection was present in a given network, BotProbe successfully detected the bot. When the total number of devices was 100 as opposed to 10 in traditional networks, BotProbe was able to produce a mean TPR above 0—possibly indicating that a greater volume of traffic allowed BotProbe to operate correctly. The equivalent IoT scenarios to which BotProbe was applied also rendered a mean TPR above 0, where it failed to make any detections in their traditional counterparts. The volume of traffic in the IoT scenarios was greater than that in the traditional equivalents, representing the more autonomous activities of IoT sensors as opposed to the more fluctuating human-like activities represented in the traditional scenarios. Although BotProbe did not detect all bots, particularly when more than a single bot was present, it did correctly identify all non-bot hosts.

BotHunter was unable to detect any bots across all scenarios, both simulated and externally acquired. It is possible that BotHunter’s approach was designed for situations where a given bot’s full lifecycle can be reported and modelled—something that may not have been available in the simulations or external datasets. The simulations did not necessarily include the infection stage, for example, though it is apparent that some of the CnC activity was at least alerted when the IRC protocol was present. When the IRC protocol was not present, BotHunter had no alerts to model at all. It should also be noted that not all botnets allow detection during the infection stage, such as when backdoors are present on a maliciously modified ISO [48] or installed at some stage of a supply chain prior to purchase [49]. BotHunter’s modelling approach may have been too rigid for the scenarios presented while also failing to make any signature detections for non-IRC bots.

Comparing each technique, it can be observed that BotMiner and BotProbe have strengths that could potentially mitigate the other’s limitations. Some limitations of BotProbe were apparent prior to experimentation, limited to the protocol that the initial filter is configured for. While BotProbe was able to detect at least one bot in most scenarios, BotProbe failed to detect every bot. BotMiner, in contrast, was able to detect all bots and is completely protocol-independent. However, BotMiner registered multiple false positives when aberrant activity was present. BotProbe did not produce any false positives, even when aberrant activity was present. Given that BotMiner is protocol-independent and was able to detect all bots in all experimental scenarios and that BotProbe did not produce any false positives, it is possible that the techniques could be used to complement one another. If the CnC command traffic can be identified from BotMiner’s netflow and alert cluster correlation, BotProbe could be used to refine BotMiner’s findings.

Whereas BotMiner and BotProbe could be complementary to one another, BotHunter was unable to detect any bots. Like BotMiner, it appears that BotHunter may have been more capable when applied to IRC-based bots, given that its IDS component was able to make some detections when the IRC protocol was used. However, unlike BotMiner, BotHunter was unable to utilise these IDS detections to render a detection. It is possible that if BotHunter’s modelling were more accommodating to modern threat scenarios or if the IDS component was made to be more sensitive, BotHunter could have been more comparable to BotMiner: they both utilise IDS and are both protocol-independent. Whether or not BotHunter would have produced false positives if this had been the case cannot be determined from the experimental results.

With only one of the three detection techniques failing to detect any bots and with certain limitations appearing to be unaffected, or sometimes even partially mitigated, by IoT scenarios, it can be concluded that traditional botnet detection approaches found in the literature, particularly BotMiner and BotProbe, are capable of detecting IoT-based botnets. While IoT-based botnets may present a serious threat in comparison to their traditional counterparts by their numbers, availability, and relative lack of security, there are techniques capable of detecting them.

## 7. Conclusions

From the results of each of the chosen botnet detection techniques observed, as applied to the presented experimental scenarios, it can be determined that techniques designed for detecting older botnets can be capable of detecting contemporary IoT-based botnets. BotMiner and BotProbe were both able to detect both traditional and IoT-based bots. BotHunter was the only technique that was unable to detect bots in any of the scenarios, both traditional and IoT scenarios. The results appear to indicate that whether a botnet is IoT-based or not will not necessarily affect the performance of any of the selected detection techniques. While the primary purpose of this work was to determine whether older techniques could work for newer IoT threats, some other potential points of investigation were found. It is apparent that the techniques examined are far better suited to detecting botnets that utilise the IRC protocol, with BotProbe being almost explicitly designed to do so. Further work should be conducted towards making all of these techniques protocol-independent.

### Future Work

Future work could include utilising the strengths of both BotMiner and BotProbe to mitigate the limitations of both. While BotMiner did perform better on IRC-based botnets, it was also capable of detecting all other bots, regardless of the protocol. Potentially utilising BotMiner in place of BotProbe’s IRC filter may allow BotProbe to then test BotMiner’s findings, which resulted in a number of false positives among the results, to reduce false reports. This approach would still only work on push-based botnets but would allow BotMiner’s high sensitivity to be tempered by BotProbe’s greater specificity.

## Figures and Tables

**Table 1:** The mean TPR and mean TNR produced by BotMiner when applied to each simulated traditional botnet scenario.

Label	Botnet	Total Devices	Infected	Aberrant	TPR	TNR
1	HTTP	10	1	0	1	1
2	HTTP	10	1	1	1	0.888889
3	HTTP	100	1	0	1	1
4	HTTP	100	1	1	1	0.989899
5	HTTP	10	3	0	1	1
6	HTTP	10	3	1	1	0.888889
7	HTTP	100	3	0	1	1
8	HTTP	100	3	1	1	0.989899
9	IRC	10	1	0	1	1
10	IRC	10	1	1	1	0.955556
11	IRC	100	1	0	1	1
12	IRC	100	1	1	1	0.99596
13	IRC	10	3	0	1	1
14	IRC	10	3	1	1	0.914286
15	IRC	100	3	0	1	1
16	IRC	100	3	1	1	0.991753
17	None	10	0	0		1
18	None	10	0	1		0.9
19	None	100	0	0		1
20	None	100	0	1		0.99

**Table 2:** The mean TPR and mean TNR produced by BotMiner when applied to each simulated IoT botnet scenario.

Label	Botnet	Total Devices	Infected	Aberrant	TPR	TNR
1	BS	10	1	0	1	1
2	BS	10	1	1	1	0.888889
3	BS	100	1	0	1	1
4	BS	100	1	1	1	0.989899
5	BS	500	1	0	1	1
6	BS	500	1	1	1	0.997996
7	BS	10	3	0	1	1
8	BS	10	3	1	1	0.857143
9	BS	100	3	0	1	1
10	BS	100	3	1	1	0.989691
11	BS	500	3	0	1	1
12	BS	500	3	1	1	0.997988
13	IRC	10	1	0	1	1
14	IRC	10	1	1	1	0.977778
15	IRC	100	1	0	1	1
16	IRC	100	1	1	1	1
17	IRC	500	1	0	1	1
18	IRC	500	1	1	1	1
19	IRC	10	3	0	1	1
20	IRC	10	3	1	1	1
21	IRC	100	3	0	1	1
22	IRC	100	3	1	1	0.997938
23	IRC	500	3	0	1	1
24	IRC	500	3	1	1	0.999598
25	None	10	0	0		1
26	None	10	0	1		0.9
27	None	100	0	0		1
28	None	100	0	1		0.99
29	None	500	0	0		1
30	None	500	0	1		0.998004

**Table 3:** The true positives, false positives, and true negatives produced by BotMiner when applied to each externally sourced dataset.

Type	Total Devices	Infected	TP	FP	TN
Trad.	10	1	1	0	9
IoT	10	1	1	4	5

**Table 4:** The mean TPR and mean TNR produced by BotProbe when applied to each simulated traditional botnet scenario.

Label	Botnet	Total Devices	Infected	Aberrant	TPR	TNR
1	IRC	10	1	0	1	1
2	IRC	10	1	1	1	1
3	IRC	100	1	0	1	1
4	IRC	100	1	1	1	1
5	IRC	10	3	0	0	1
6	IRC	10	3	1	0	1
7	IRC	100	3	0	0.666667	1
8	IRC	100	3	1	0.466667	1

**Table 5:** The mean TPR and mean TNR produced by BotProbe when applied to each simulated IoT botnet scenario.

Label	Botnet	Total Devices	Infected	Aberrant	TPR	TNR
1	IRC	10	1	0	1	1
2	IRC	10	1	1	1	1
3	IRC	100	1	0	1	1
4	IRC	100	1	1	1	1
5	IRC	500	1	0	1	1
6	IRC	500	1	1	1	1
7	IRC	10	3	0	0.46667	1
8	IRC	10	3	1	0.46667	1
9	IRC	100	3	0	0.6	1
10	IRC	100	3	1	0.8	1
11	IRC	500	3	0	0.6	1
12	IRC	500	3	1	0.46667	1

**Table 6:** The mean TPR (from IDS and overall) and mean TNR produced by BotHunter when applied to each simulated traditional botnet scenario.

Botnet	Total Devices	Infected	Aberrant	IDS TPR	TPR	TNR
HTTP	10	1	0	0	0	1
HTTP	10	3	0	0	0	1
HTTP	10	1	1	0	0	1
HTTP	10	3	1	0	0	1
HTTP	10	0	0			1
HTTP	100	1	0	0	0	1
HTTP	100	3	0	0	0	1
HTTP	100	1	1	0	0	1
HTTP	100	3	1	0	0	1
HTTP	100	0	0			1
IRC	10	1	0	1	0	1
IRC	10	3	0	1	0	1
IRC	10	1	1	1	0	1
IRC	10	3	1	1	0	1
IRC	10	0	0			1
IRC	100	1	0	1	0	1
IRC	100	3	0	1	0	1
IRC	100	1	1	1	0	1
IRC	100	3	1	1	0	1
IRC	100	0	0			1

**Table 7:** The mean TPR (from IDS and overall) and mean TNR produced by BotHunter when applied to each simulated IoT botnet scenario.

Botnet	Total Devices	Infected	Aberrant	IDS TPR	TPR	TNR
BS	10	1	0	0	0	1
BS	10	3	0	0	0	1
BS	10	1	1	0	0	1
BS	10	3	1	0	0	1
BS	10	0	0			1
BS	100	1	0	0	0	1
BS	100	3	0	0	0	1
BS	100	1	1	0	0	1
BS	100	3	1	0	0	1
BS	100	0	0			1
BS	500	1	0	0	0	1
BS	500	3	0	0	0	1
BS	500	1	1	0	0	1
BS	500	3	1	0	0	1
BS	500	0	0			1
IRC	10	1	0	1	0	1
IRC	10	3	0	1	0	1
IRC	10	1	1	1	0	1
IRC	10	3	1	1	0	1
IRC	10	0	0			1
IRC	100	1	0	1	0	1
IRC	100	3	0	1	0	1
IRC	100	1	1	1	0	1
IRC	100	3	1	1	0	1
IRC	100	0	0			1
IRC	500	1	0	1	0	1
IRC	500	3	0	1	0	1
IRC	500	1	1	1	0	1
IRC	500	3	1	1	0	1
IRC	500	0	0			1

**Table 8:** The true positives (from IDS and overall), false positives (from IDS and overall), true negatives, and false negatives produced by BotHunter when applied to each externally sourced dataset.

Type	Total Devices	Infected	IDS TP	IDS FP	TP	FP	TN	FN
Trad.	10	1	0	0	0	0	9	1
IoT	10	1	0	0	0	0	9	1

**Table 9:** Each hypothesis addressed for the traditional botnet detection techniques.

Detection Technique	H_1_	H_2_	H_3_	H_4_	H_5_	H_6_
BotMiner	rejected	accepted	accepted	rejected	rejected	accepted
BotProbe	rejected	accepted	rejected	accepted	accepted	rejected
BotHunter	accepted	rejected	rejected	accepted	accepted	rejected

## Data Availability

Data are contained within the article.

## Abbreviations

The following abbreviations are used in this manuscript:

IoT	Internet of Things
CnC	Command and control
IRC	Internet Relay Chat
P2P	Peer-to-Peer
HTTP	Hypertext Transfer Protocol
DDoS	Distributed Denial of Service
TPR	True positive rate
TNR	True negative rate
IDS	Intrusion Detection System
fph	Flows per hour
ppf	Packets per flow
bpp	Average bytes per packet
bps	Average bytes per second
DNS	Domain Name Service
SDN	Software-Defined Network
AMQP	Advanced Message Queuing Protocol
TCP	Transmission Control Protocol
BS	Bitstream
pcap	Packet capture
